# Methods to Compare Predicted and Observed Phosphene Experience in tACS Subjects

**DOI:** 10.1155/2018/8525706

**Published:** 2018-12-06

**Authors:** Aprinda Indahlastari, Aditya K. Kasinadhuni, Christopher Saar, Kevin Castellano, Bakir Mousa, Munish Chauhan, Thomas H. Mareci, Rosalind J. Sadleir

**Affiliations:** ^1^School of Biological and Health Systems Engineering, Arizona State University, USA; ^2^Department of Clinical and Health Psychology, Center for Cognitive Aging and Memory, McKnight Brain Institute, University of Florida, USA; ^3^Department of Biomedical Engineering, University of Florida, USA; ^4^GE Healthcare, Waukesha, WI, USA; ^5^Department of Biochemistry and Molecular Biology, University of Florida, USA

## Abstract

**Background:**

Phosphene generation is an objective physical measure of potential transcranial alternating current stimulation (tACS) biological side effects. Interpretations from phosphene analysis can serve as a first step in understanding underlying mechanisms of tACS in healthy human subjects and assist validation of computational models.

**Objective/Hypothesis:**

This preliminary study introduces and tests methods to analyze predicted phosphene occurrence using computational head models constructed from tACS recipients against verbal testimonies of phosphene sensations. Predicted current densities in the eyes and the occipital lobe were also verified against previously published threshold values for phosphenes.

**Methods:**

Six healthy subjects underwent 10 Hz tACS while being imaged in an MRI scanner. Two different electrode montages, T7-T8 and Fpz-Oz, were used. Subject ratings of phosphene experience were collected during tACS and compared against current density distributions predicted in eye and occipital lobe regions of interest (ROIs) determined for each subject. Calculated median current densities in each ROI were compared to minimum thresholds for phosphene generation.

**Main Results:**

All subjects reported phosphenes, and predicted median current densities in ROIs exceeded minimum thresholds for phosphenes found in the literature. Higher current densities in the eyes were consistently associated with decreased phosphene generation for the Fpz-Oz montage. There was an overall positive association between phosphene perceptions and current densities in the occipital lobe.

**Conclusions:**

These methods may have promise for predicting phosphene generation using data collected during in-scanner tACS sessions and may enable better understanding of phosphene origin. Additional empirical data in a larger cohort is required to fully test the robustness of the proposed methods. Future studies should include additional montages that could dissociate retinal and occipital stimulation.

## 1. Introduction

Transcranial alternating current stimulation (tACS) is a noninvasive technique used to modulate brain function [1]. Configurations used for tACS typically employ a pair of large electrodes (~35 cm2) that are placed close to presumed target brain areas [2]. The underlying mechanism of tACS is believed to be that application of external oscillating current to the cortex can induce neural entrainment at the stimulating frequency [3, 4]. Helfrich et al. [5] were the first to confirm tACS-induced neural entrainment in humans by showing tACS-related increases of alpha EEG activity in the posterior cortex were associated with enhanced visual target detection. The stimulation effects of tACS are assumed to be correlated with subthreshold changes in cortical electric fields [6]. Radman et al. [7] reported that the minimum electric field magnitude required to depolarize pyramidal cells was in the range of 28–79 V/m. These values were obtained from *in vitro* whole-cell recordings in rat brain slices. Since these pyramidal neurons were inactive during *in vitro* measurements, the threshold for depolarization of active pyramidal cells *in vivo* may be lower [8]. A recent study by Vöröslakos et al. [9] reported a voltage gradient of at least 1 V/m was required to initiate neuronal spiking based on an intracellular and extracellular recording in rats.

Phosphenes are light sensations occurring in the eyes that can be induced by external electrical stimuli [10]. Previous studies have found that phosphene generation often occurs in subjects undergoing tACS [11]. Different tACS frequencies elicit different perceived phosphene intensity levels [4]. Stimulation frequencies above 8 Hz result in increased phosphene intensity, with the most intense levels observed at around 20 Hz [12]. Several papers have suggested the visual cortex or the retina as the possible origin of phosphenes [3, 13–17].

A local and “purely” cortical TMS application over the visual cortex was found to also induce phosphenes and, in fact, was used to measure the effects of tDCS on visual cortex excitability [3]. This observation suggested that phosphenes could originate in the visual cortex (cortical phosphenes). Kanai et al. [18] performed tACS over the visual cortex using an Oz- (occiput-) Cz (vertex) montage, and observed phosphene events when subjects were stimulated at 20 Hz in bright conditions and 10 Hz in dark conditions. These frequencies corresponded to those of EEG beta (12–30 Hz) and alpha (8–12 Hz) waves [19]. Therefore, tACS was assumed to generate cortical phosphenes by interacting with specific frequencies of cortical activity in the visual cortex [18]. Further, tACS at 20 Hz administered via an Oz-Cz montage was found to lower the TMS-induced phosphene threshold while Fpz- (nasion-) Cz montage did not [14]. Therefore, the study by Kanai et al. [14] concluded locating electrodes further away from the occipital lobe increased phosphene generation thresholds. Bosking et al. [20] found that there was a saturation in phosphene size generated by electrical stimulation via implanted subdural electrodes in the visual cortex. This saturation implied that the functional network of the cortex might impose restrictions on the current spread of artificially evoked activity.

Other reports have suggested that phosphene perception in tACS subjects, including those who undergo visual cortex stimulation, may actually originate in the retina. The ability of the retina to elicit phosphenes was first introduced by Brindley [21], where direct retinal stimulation was performed using conjunctival electrodes. Schutter and Hortensius [16] reported that tACS in subjects using Oz-Cz and Fpz-Cz montages induced phosphenes and found recorded voltages near the eyes increased during the stimulation with either electrode placement [3, 16]. However, subjects perceived more intense phosphenes with the Fpz-Cz montage [16]. Therefore, placing tACS electrodes closer to the eyes was found to increase phosphene intensity in subjects during stimulation, suggesting retinal phosphene generation via current “leakage” via the scalp to the retina.

The occurrence of cortical phosphenes proposed by Kanai et al. [18] was later challenged by Schwiedrzik [19], who claimed that the frequency-dependent effects in bright and dark conditions were controlled by dark adaptation of the retina, instead of neural activation in the visual cortex. Kar and Krekelberg [15] supported this claim by showing that neuronal activity in retinal ganglion cells peaked at 20 Hz in lighted conditions and at 10 Hz in dark conditions. In addition, Kar and Krekelberg [15] showed that a single 1 mA pulse applied over the visual cortex could elicit phosphenes and that ongoing cortical oscillations were not required to evoke phosphenes. Laakso and Hirata [17] later performed a computational study to confirm that current density magnitudes found in the eyes as a result of visual cortex stimulation were large enough to elicit retinal phosphenes [17].

To date, there has been no study that investigates phosphene perception by directly comparing tACS subject testimonies and predictions derived from subject-specific models. Such a study would be particularly useful, since exact electrode locations and subject-specific head geometries are modeled, thus producing more precise predictions of current flow and likely phosphene experience. In this study, we propose methods to compare predicted phosphene occurrence and subject verbal testimonies of phosphene experience in tACS recipients. Predicted current density distributions in the eyes and the occipital lobe were calculated using computational head models derived from six tACS recipient MR image datasets. Predicted values were then compared to previously published thresholds for phosphene generation [3, 7, 15, 17, 22, 23] and verified against subject phosphene testimonies. We suggest that the methods described in this paper may be of use in future investigations into the origins of phosphene perception.

## 2. Materials and Methods

All experimental procedures reported in this study followed protocols approved by the University of Florida (UF) and Arizona State University (ASU) Institutional Review Boards. Six healthy participants (mean age 24, range 20–39 years old) received tACS-like treatment at 131 approximately 10 Hz as part of our previously reported magnetic resonance electrical impedance tomography (MREIT) current density and tissue conductivity imaging studies [24, 25]. High- resolution T1- and diffusion-weighted images, and phosphene testimonies, were gathered as part of the overall study protocol. Following subject consent, but prior to study interventions, each subject completed a minimental state examination (MMSE) to eliminate neurological disorders and an Edinburgh Inventory test to confirm their right-handedness. Participant T1-weighted structural data was segmented and combined with coregistered white matter diffusion tensors to define anisotropic conductivity finite element models. Each subject-specific model comprised 10 different tissue compartments. Eyes and occipital lobes were defined as regions of interest (ROIs), and current densities and electric fields within them were assessed. The methodology and dataset used in this study are available to share upon request.

### 2.1. Electrode Placements

**Figure 1:**
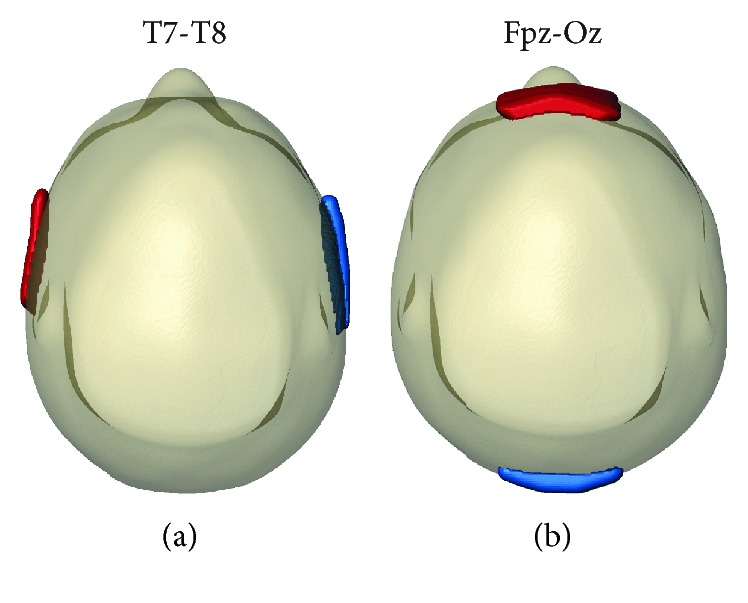
Electrode configurations placed on tACS recipients. Montages used were (a) T7-T8 and (b) Fpz-Oz. First named electrodes were anodes (red) and second named electrodes were cathodes (blue). Here, anodes denote electrodes used for initial positive pulses.

Two electrode montages were used, namely, T7-T8 and Fpz-Oz, as shown in Figure 1. Carbon electrodes (neuroConn, Ilmenau, Germany) with an area of 25 cm^2^ were encapsulated in saline-soaked sponges and secured on participant heads using an elastic bandage (Vetrap, 3M).

### 2.2. Imaging Sequences and Parameters

T1- and diffusion-weighted images of participants were acquired using a 3T MRI Phillips Achieva scanner housed at the Advanced Magnetic Resonance Imaging and Spectroscopy Facility, UF McKnight Brain Institute. The high-resolution 3D FLASH T1-weighted datasets were acquired with a 240 × 240 matrix size (voxel dimension of 1 mm^3^) over 160 sagittal slices, each of 1 mm thickness. High angular resolution diffusion-weighted imaging (HARDI) datasets were acquired over 6 gradient directions with low b-value (100 s/mm^2^) and a total of 64 gradient directions with high b-value (1000 s/mm^2^). Two sets of 6-direction DWI data were acquired, each having opposite phase encoding direction and were combined using the command topup in FSL [26]. The resulting diffusion-weighted images comprised 70 sagittal slices of 112 × 112 matrix size at 2 mm^3^ isotropic resolution. T1-weighted and HARDI datasets in each subject were coregistered prior to FEM construction and model tissue conductivity assignments.

### 2.3. tACS Procedures and Subject Testimonies

**Figure 2:**
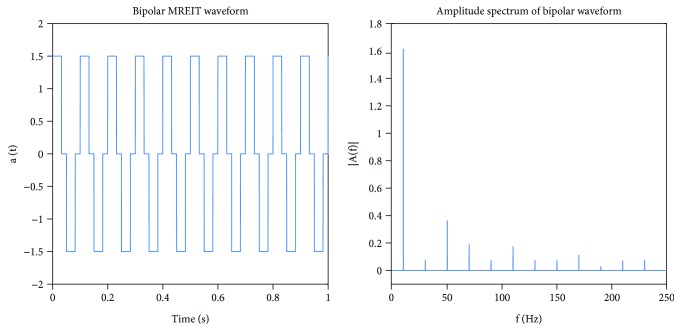
Applied current waveform and corresponding frequency distribution. The current waveform used in this study was bipolar (10 Hz) and had an amplitude of 1.5 mA.

Current administrations were performed following the MREIT-current density imaging (CDI) procedures described in our previous study [24]. The MREIT-CDI sequence applied a series of alternating, bipolar pulses with amplitudes of 1.5 mA. Bipolar stimulation comprised cycles of alternating positive and negative 32 ms pulses (Figure 2). The stimulation waveforms were implemented as a series of rectangular pulses to allow synchronization with TR during MR image acquisition. Each current pulse occupied 32 ms of each 50 ms TR, and thus, the duty cycle was approximately 64%. Frequency spectra were obtained by fast Fourier transform showed peaks at 10 Hz, respectively (Figure 2).

Verbal testimonies of subject phosphene perceptions were recorded during MREIT imaging procedures, while subjects were still within the MR scanner with the room lights off in all sessions. All participants were asked to rate the intensity levels of phosphenes on a scale from 1 to 10, with 1 being no phosphenes and 10 being a white field. Averaged ratings were used if subjects underwent multiple stimulation procedures using the same montage.

### 2.4. Head Model Construction and Tissue Segmentation

A realistic head model was constructed for each participant. Each model comprised ten different tissue types and nine different conductivities. Prior to segmentation, structural T1-weighted datasets were resampled to 256 × 256 × 256 1 mm^3^ isotropic resolution using FreeSurfer (Cambridge, MA). Details of the segmentation pipeline are contained in Indahlastari et al. [27]. The eye ROI included the sclera, lens, and the aqueous vitreous of both the left and right eyes. The aqueous vitreous was assumed to have the same conductivity as cerebrospinal fluid (CSF). The occipital cortex ROI (OCC) was automatically segmented in FreeSurfer and manually corrected in ScanIP (Simpleware, Synopsys Inc., Exeter, UK) using a human atlas as a reference [28].

### 2.5. Finite Element Simulation

Segmented tissue masks were then processed following the modeling pipeline defined in our previous work [24]. The resulting segmented model was meshed using ScanFE (Simpleware, Synopsys Inc., Exeter, UK) to produce meshes with approximately 20 million tetrahedral elements. The meshed head models were imported into COMSOL (COMSOL Inc., Burlingham, MA, USA) and literature-referenced tissue conductivity values typical of frequencies under 1 kHz were assigned (Table 1). Isotropic and anisotropic head models were distinguished by how conductivity values in white matter compartments were assigned. In anisotropic models, white matter conductivity tensor distributions were interpolated using principal eigenvectors of diffusion tensor images and assumptions of white matter longitudinal and transverse conductivities. In isotropic models, a conductivity of 0.3835 S/m was assigned to white matter compartments. Further details on the white matter conductivity tensor formulation and the forward model calculation may be found in Indahlastari et al. [29].

The Laplace equation was solved on each model. Boundary conditions were specified as input normal current densities (*J*_input_) of 0.4 mA/m^2^ applied to anode electrodes (T7, Fpz), while cathode electrodes (T8 or Oz, respectively) were set to ground voltage. Voltage values throughout the entire head volume were solved for using COMSOL. Derived current density magnitudes were then interpolated onto a 256 × 256 × 256 mm grid using the MATLAB-COMSOL Livelink Interface (MLI) and masked to eye or occipital lobe ROIs. Median current densities in ROIs were computed in MATLAB (Mathworks, Natick, MA) and compared to literature-sourced values for the minimum thresholds to induce phosphenes [17, 22]. Discrepancies in median current densities between isotropic and anisotropic models were calculated with respect to sotropic models. To clearly compare the effects of each electrode montage on current density distributions in ROIs, median current densities were expressed as the percentage ratio (%J) of median current density magnitude (*J*_med_) in each ROI to the input current density (*J*_input_) in each simulation case.

A nonparametric local-linear regression model was used to analyze correspondence between both raw and logged current density distributions in the eye, medial walls of the occipital lobe and the remainder of the occipital lobe, and subject ratings. An Epanechnikov kernel was used and 50 bootstrap replications were tested. Logged data were used because of the large dynamic range of the current density data [30]. Additional ROIs with surface areas of around 25 mm^2^ and volumes averaging around 565 mm^3^ were sampled manually from the medial walls of each participant's OCC ROI. Coefficients of determination (R^2^ values) and slopes were estimated using the npregress command in Stata (StataCorp LP, College Station, TX).

Finally, predicted electric field magnitudes were computed from median current densities observed in the OCC ROI divided by gray matter conductivity found in Table 1. These electric fields were compared against literature-sourced values for electric field thresholds required to depolarize pyramidal cells in the cortex [3, 7, 9].

## 3. Results

Overall, current density magnitudes in the eye ROI were about three times as large as in the OCC. Discrepancies in median current densities between anisotropic and isotropic models were within ±17% in the EYE and ± 30% in OCC ROI. Details of study results are contained in the following subsections.

### 3.1. Subject Phosphene Testimonies

Table 2 summarizes subject phosphene ratings. All subjects observed phosphenes in peripheral visual fields during the tACS-like procedures. Stimulation using the Fpz-Oz montage received the highest average phosphene ratings with an average of 6 (range 4–7). The average rating for the T7-T8 montage was 3.5 (range 2–4).

### 3.2. Heat Maps of Current Density Distributions in ROIs

**Figure 3:**
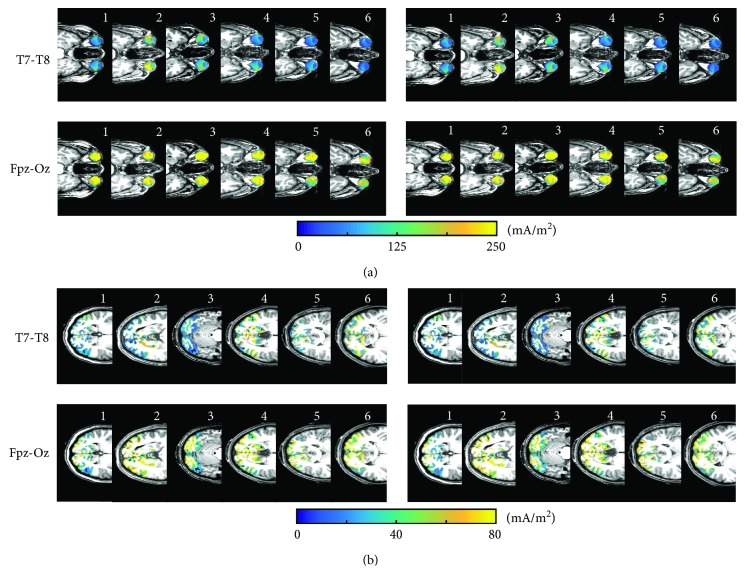
Predicted current density distributions in the central axial slice of individual ROIs. Images showing simulated current density maps for isotropic (left) and anisotropic (right) models in (a) EYE and (b) occipital lobe (OCC) ROIs. Subject numbers are shown in the top right corner of each current density image.

Figure 3 shows predicted current density distributions in the central axial slices of both ROIs. Current density distributions in ROIs exhibited similar patterns in both isotropic and anisotropic models. The largest current density values in both ROIs were observed for the Fpz-Oz montage. Fpz-Oz and T7-T8 montages produced approximately equal current density distributions in the left and right eyes.

### 3.3. Median Current Densities in ROIs

**Figure 4:**
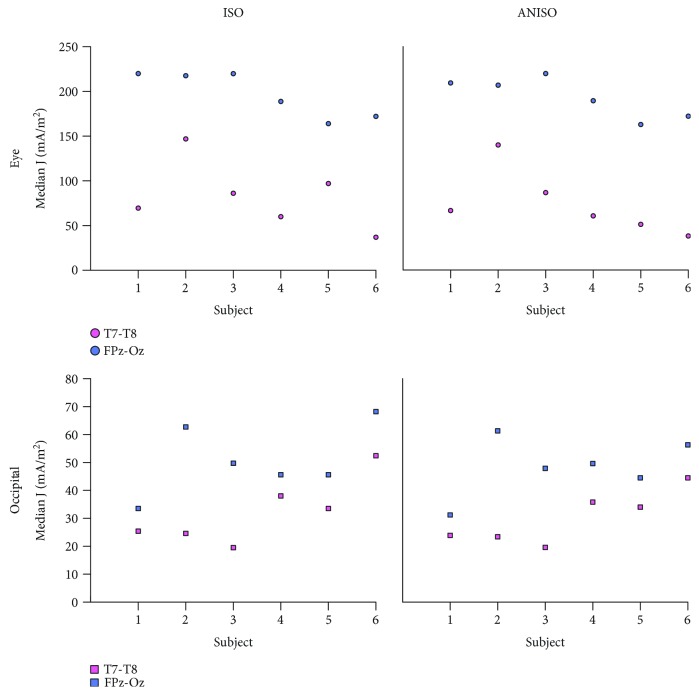
Calculated median current density values in ROIs. All values exceeded the threshold current density assumed to induce phosphenes (1 mA/m^2^) [22].

Figure 4 shows plots of median current density values in the EYE and OCC ROIs for both isotropic and anisotropic models. All calculated median current density values in ROIs exceeded the 1 mA/m^2^ threshold presumed for phosphene generation [22]. The largest median current densities in the eyes for both isotropic and anisotropic models were 220 mA/m^2^ in the EYE and 68 mA/m^2^ in the OCC ROIs, respectively. Models of the Fpz-Oz montage had the largest median current densities in both ROIs. The smallest median current densities in the EYE and OCC ROIs were from models with the T7-T8 montage.

Median current density values in ROIs, expressed as a percentage of input current density (%Js), are shown in Table 3. On average, the median eye ROI current density was 9–55% of input current densities, while the OCC ROI were around 3–17%, across all electrode configurations for both isotropic and anisotropic models. In the EYE and OCC ROIs, %J values were found larger in Fpz-Oz than in T7-T8 montages, for both isotropic and anisotropic models.

**Figure 5:**
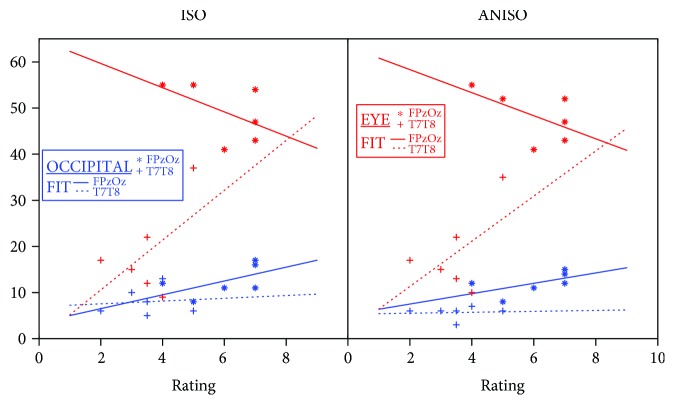
Scatter plots comparing subject ratings and median current density percentages (%Js) for both Fpz-Oz and T7-T8 montages for (left) isotropic and (right) anisotropic models. Fit lines shown are to median data and illustrate trends only.

Table 4 summarizes findings from the nonparametric regression model used to examine the dependence of all logged current densities in the medial occipital lobe, the remainder of the occipital lobe, and eye compartments upon subject ratings for each montage. The R^2^ values found for T7-T8 models using both logged and unlogged data were over 0.4 for both isotropic and anisotropic model data. The model therefore suggested a large percentage of subject ratings was explained by current densities in eye and occipital lobes. Coefficients found in T7-T8 montage models suggested a stronger positive dependence of ratings on current densities in the eye than on occipital lobe current densities. Ratings for this montage also depended more on occipital medial wall current densities than on current densities in the remainder of the occipital lobe. For the Fpz-Oz montage, relationships between current densities in the eyes and occipital lobe and subject ratings were not as well correlated, with models showing lower calculated R^2^ values (0.38 for logged anisotropic model data and 0.33 for logged isotropic model data). For both isotropic and anisotropic models, there was a negative association between logged current densities in the eye and subject ratings and positive associations between current densities in the occipital lobe and ratings, as shown in Figure 5.

### 3.4. Electric Field Current Densities in the Occipital Cortex

Table 5 shows norms of electric fields computed in OCC ROIs. Computed electric fields in both tissue anisotropy models for T7-T8 and Fpz-Oz montages were in the range of 0.20–0.68 V/m and below the estimated threshold for depolarization of pyramidal neurons in the visual cortex [7, 9].

## 4. Discussion

In this study, we tested our proposed methods to compare predicted and observed phosphenes in six healthy tACS recipients. We expected that phosphenes would be experienced by all subjects, since tACS at 10 Hz has been repeatedly been shown to induce phosphenes even at low current intensities [12]. All predicted median current densities in ROIs, calculated using either isotropic or anisotropic models, exceeded threshold current densities reported to cause phosphenes [22]. In the following subsections, we discuss the context of study results. Observations are applicable to both isotropic and anisotropic models, unless specified otherwise.

### 4.1. Modeling Predictions and Subject Testimonies

The Fpz-Oz montage had the highest phosphene ratings from subjects and the largest %Js in both ROIs, while the other montage ratings were lower, showing a broad agreement between model predictions and testimonies (Figure 5). An interesting occurrence was observed for subject 7 and the T7-T8 montage. Subject 7 scored this montage with a much higher phosphene rating (5) than average (3.5). This finding was consistent with predicted current densities in the eye ROIs for this subject that showed large median current densities for both isotropic and anisotropic models. The predicted %J in the T7-T8 model of subject 7 was 37% (average = 19%) for the isotropic model and 35% (average = 18%) for the anisotropic model. However, calculated %Js in the OCC ROI of the subject 7 T7-T8 models had similar values to group averages (6%, with subject 7 having an average of 8% for the isotropic model, and 6%, with subject 7 having an average of 6% for the anisotropic model). Therefore, the high phosphene rating given by subject 7 to the T7-T8 montage might have been related to large current densities in the eyes, suggesting retinal phosphenes. Results of nonparametric regression analysis showed moderate R^2^ values (0.33–0.50) in all the four models suggesting a correlation between logged current density norms in eyes and occipital lobe compartments and phosphene intensity reports. The fact that predicted current densities in the eyes were negatively associated with phosphene observations may indicate that the eye response to phosphenes is saturated beyond a certain current intensity. We caution that these results are preliminary because of the limited dataset used. However, we did observe that nonparametric regression produced higher coefficients of regression (R^2^) values, but the same overall trends, as we found when performing linear regression on the logged data. We recommend that nonparametric statistical tests be used with a larger sample size to produce a more robust analysis because of the large ranges and likely nonlinear dependence of subject ratings on current density distributions.

All subjects in this study reported phosphene occurrence in their peripheral visual fields. The medial anterior wall of the visual cortex has been associated with the peripheral areas of the visual field [18]. Coefficients related to the medial wall of the occipital lobe averaged around 0.23 for both the T7-T8 and Fpz-Oz montages. For T7-T8 models, coefficients for the medial occipital wall were stronger than for those in the remainder of the occipital lobe; however, there was a large overlap between coefficients. For the isotropic Fpz-Oz montage model, there was a much larger association between rating and occipital lobe remainder than for the medial wall, which may indicate a limitation in the isotropic model or the limitations of the regression analysis for this sample. We caution that these observations cannot be considered conclusive because of the lack of scale normalization in phosphene response and the low number of participants.

### 4.2. The Origins of Phosphenes

Previous studies have suggested that tACS application via electrode locations near the eyes could elicit retinal phosphenes via current leakage from the scalp to the eyes [16, 19]. Therefore, phosphenes observed in subjects stimulated with Fpz-Oz configuration might have originated in the retina. Predicted electric field values in OCC ROIs suggested that both montages did not produce high enough electric fields that may influence pyramidal cell depolarization in the visual cortex. This preliminary finding suggests none of the subjects would have experienced cortical phosphenes, leaving the possibility of retinal phosphenes. This observation was further supported by findings in subject 7 with T7-T8 montage and the correlation plots (Figure 5). The slope of the linear fit between subject ratings and %Js in the eye ROIs was the absolute largest for T7-T8 placement.

Neuronal cell orientation can affect the likelihood of neuronal activation and may contribute to phosphene generation [31]. Electrical stimulation applied in the radial direction to the retina was found to be more sensitive to induce retinal phosphenes due to the preferred orientation of retinal cells [21]. A modeling study by Laakso and Hirata [17] confirmed that current flow entering the eyes from a nearby transcranial electrode (Fpz) was in the radial direction to the eyes. In the human cortex, another computational study [6] showed the electrical fields introduced by transcranial electrodes (M1-contralateral supraorbital) were primarily tangential to the cortical surface in brain regions directly underneath the electrodes. The pyramidal cell bodies (soma) that are the most sensitive to membrane depolarization are located in the inner layer of the cortex [7] with the somadendritic axis oriented perpendicular to the cortical surface [6, 32]. Therefore, in our case of montage FPz-Oz, transcranial currents are more likely to activate neurons in the retina than in the cortex, suggesting the likelihood of phosphene generation in the retina.

### 4.3. Modeling Limitations

We recognize limitations in computational models used in our study. Segmenting ROIs incorrectly, for instance, might alter predicted current density distributions in the ROIs. We computed the percentage differences between calculated median current densities in modified ROIs and reported values in Figure 4. Modified ROIs involved application of either an isotropic one pixel dilation or erosion process in Simpleware. Dilated and eroded eye ROIs produced an average of 10% and 7% difference, respectively, across montages and tissue anisotropy assignments. For OCC ROIs, dilated and eroded regions were different by 17% and 7%, respectively, in all models. Computed percentage differences in median current densities in dilated and eroded ROIs were mainly contributed by CSF compartments with the largest conductivity value. The eye ROIs had a large content of CSF while the OCC ROIs interfacing with CSF compartments on the surface. All computed median current densities in dilated and eroded ROIs exceeded the thresholds for phosphenes and thus any inaccuracy in ROIs segmentation would not affect the modeling outcomes in this study. Moreover, electrode montages modeled in this study were limited to available montages used in *in vivo* tACS current density imaging studies [24, 25]. These montages were either involving both electrodes near the eyes and visual cortex (Fpz-Oz) or further away from these structures (T7-T8) and thus limiting our analysis of electrode proximity to the occipital lobe and phosphene generation.

### 4.4. Future Studies

The results presented in this study demonstrated our proposed methods of using computational modeling and verbal testimonies as useful to assess phosphene perception in tACS recipients. These methods should be implemented in a larger cohort to test the robustness of our statistical findings. In order to separate the possible relationship between electrode location and cortical or retinal phosphenes, future tACS studies should include electrode montages with both the anode and cathode electrodes located near the occipital cortex, or with both near the eyes. In addition, the association between the possible origins of phosphenes and the likelihood of neuron firing in that origin can be explored by computing additional parameters. For instance, calculating the radial components of current density entering the eye compartments and both the tangential and normal electric fields on the surface of the occipital cortex. Neuron firing may also depend on the input from the neighboring cells [15]. For instance, signal conduction from the photoreceptors to the ganglion cells may contribute to retinal activation and thus lighting condition alone may affect the likelihood of retinal phosphenes. Therefore, lighting conditions during stimulation should be closely monitored to ensure consistency across subjects. Subject testimony of phosphene perception should include a more detailed questionnaire, including reporting whether the eyes were closed or opened during stimulation, identifying phosphene presence in the left or right eye or both, and the frequency of light-flash perceptions. Finally, subject phosphene ratings should be normalized by the use of a standardized metric [33].

## 5. Conclusion

This study presented initial results from a novel approach that compared predicted modeled current densities and reported phosphene perceptions. Predicted current densities were assessed in two ROIs associated with possible phosphene origin: the eyes and the occipital lobe. As expected, all tACS recipients stimulated at 10 Hz reported phosphene generation. Predicted median current density values in ROIs exceeded current density thresholds previously reported for phosphene generation. There was not enough evidence acquired to separate retinal and cortical phosphenes. However, our initial findings suggested that all subjects experienced retinal phosphenes from receiving tACS using T7-T8 and Fpz-Oz montages. Further studies using our proposed methods may contribute in understanding the association between electrical stimulation and phosphene perception.

## Figures and Tables

**Table 1:** Literature-referenced tissue conductivities used in anisotropic head models. Values reported here are for tissue conductivity measured below 1 kHz. Averaged values were used if multiple tissue conductivity values below 1 kHz were found. Bone conductivity was calculated using the formula *σ* = (*σ*_can_·*σ*_cor_)^1/2^ where *σ*_can_ is cancellous and *σ*_cor_ is cortical tissue conductivity value. In isotropic models, white matter conductivity was assumed to be 0.3835 S/m.

Tissue type	Conductivity values (S/m)	Reference
Air	0	—
Blood	6.7 × 10^–1^	Geddes and Baker (1967)
Bone^∗^	1.09 × 10^–2^	Akhtari et al. (2002)
Cerebrospinal fluid	1.8	Baumann et al. (1997)
Fat	2.5 × 10^–2^	Gabriel et al. (1996)
Gray matter	1.0 × 10^–1^	Gabriel et al. (1996)
Muscle	1.6 × 10^–1^	Geddes and Baker (1967)
Sclera, lens	5.0 × 10^–1^	Gabriel et al. (1996)
Skin	4.3 × 10^–1^	Holdefer et al. (2006)
White matter	1.2 × 10^0^ (long.)	Geddes and Baker (1967)
	1.2 × 10^–1^ (trans.)	

**Table 2:** Numerical ratings of phosphene perceptions by each subject. All subjects were asked to rate their phosphene perceptions from 1 to 10 with 1 being “no phosphenes” and 10 being a completely white field.

	T7-T8	Fpz-Oz
Subject 1	2	5
Subject 2	5	7
Subject 3	3.5	4
Subject 4	3	7
Subject 5	3.5	6
Subject 6	4	7
Average	3.5	6

**Table 3:** Median current density %Js in both ROIs for isotropic and anisotropic models. %Js were calculated as the ratio of median current density to input current density for each electrode configuration.

	J (%) in eyes (isotropic)		J (%) in eyes (anisotropic)	
	T7-T8	Fpz-Oz	T7-T8	Fpz-Oz

Subject 1	17	55	17	52
Subject 2	37	54	35	52
Subject 3	22	55	22	55
Subject 4	15	47	15	47
Subject 5	12	41	13	41
Subject 6	9	43	10	43
Average	19	49	18	48

	J (%) in OCC (isotropic)		J (%) in OCC (anisotropic)	
	T7-T8	Fpz-Oz	T7-T8	Fpz-Oz

Subject 1	6	8	6	8
Subject 2	6	16	6	15
Subject 3	5	12	3	12
Subject 4	10	11	6	12
Subject 5	8	11	6	11
Subject 6	13	17	7	14
Average	8	13	6	12

**Table 4:** Coefficients of determination (R^2^) and association estimates with 95% confidence intervals for nonparametric local-linear regression model on logged current density data. All coefficients were significant (*α* < 0.05).

Montage	R^2^	Coefficients (logged data)		
		Eye	Occipital, medial wall	Occipital, remainder
Fpz-Oz, iso	0.33	−0.206 (−0.27–0.12)	0.214 (0.16, 0.27)	0.764 (0.72, 0.82)
Fpz-Oz, aniso	0.38	−0.343 (−0.45, −0.21)	0.178 (0.11, 0.23)	0.101 (0.09, 0.17)
T7-T8, iso	0.50	0.471 (0.41 0.54)	0.298 (0.24, 0.36)	−0.016 (−0.08, 0.07)
T7-T8, aniso	0.45	0.508 (0.46, 0.59)	0.222 (0.17, 0.29)	0.189 (0.16, 0.24)

**Table 5:** Predicted electric fields (EF) in the occipital lobe (OCC) for isotropic and anisotropic models. All computed electric fields were smaller than 28–79 V/m [7] or 1 V/m [9], the electric field action potential threshold for layer V pyramidal neurons and intact rat brain, respectively.

	EF (V/m) (isotropic)		EF (V/m) (anisotropic)	
	T7-T8	Fpz-Oz	T7-T8	Fpz-Oz
Subject 1	0.25	0.34	0.24	0.31
Subject 2	0.25	0.63	0.23	0.61
Subject 3	0.20	0.50	0.20	0.48
Subject 4	0.38	0.46	0.36	0.50
Subject 5	0.34	0.46	0.34	0.45
Subject 6	0.52	0.68	0.45	0.56
Average	0.32	0.51	0.30	0.48

## Data Availability

Processed data (deidentified COMSOL model data, deidentified spreadsheet data) reported in this study may be obtained by application to the authors.
